# Distinct contributions of mu opioid and CB2 cannabinoid receptors to neuroimmune and behavioral responses

**DOI:** 10.3389/adar.2026.16646

**Published:** 2026-07-29

**Authors:** Berhanu Kibret, Emmanuel S. Onaivi, Venkatanarayanan Sharma

**Affiliations:** 1 Department of Pharmaceutical Sciences, School of Pharmacy, University of Maryland Baltimore, Baltimore, MD, United States; 2 Department of Biology, William Paterson University, Wayne, NJ, United States

**Keywords:** cannabinoids, CB2, cytokines, inflammation, MOR

## Abstract

Marijuana (cannabis) and cannabinoids are getting global medical and recreational approvals in this era of the opioid epidemic. Both opiates and cannabis are often co-abused, and their use increases the risk of opioid and cannabis use disorders (OUDs and CUDs), and dependency. Opioid and cannabinoid systems share many neuromodulating and pharmacological effects by activating opioid and cannabinoid receptors, respectively. Inflammation is increasingly implicated in many diseases, and mu-opioid receptor (MOR) and CB2 cannabinoid receptor (CB2R) have been linked to neuroinflammation. The hypothesis that changes in MOR and CB2R mediated behavioral and cytokine alterations are associated with their roles in inflammation was tested here. Naïve male C57Bl/6J as wild type, MOR KO mice with deletion of MOR, DAT-*Cnr2* cKO mice with deletion of CB2R from dopamine neurons and CX3Cr1-*Cnr2* cKO mice with deletion of CB2R from microglia were used in the study. Nociception was assessed using tail-flick latency, followed by ELISA to quantify levels of cytokines and chemokines in the cerebellum and prefrontal cortex (PFC) regions of the animals. MOR KO, DAT-*Cnr2*, and CX3Cr1-*Cnr2* conditional knockout mice exhibited distinct behavioral and neuroimmune phenotypes, marked by genotype- and region-specific alterations in cytokines and chemokine. CX3Cr1-*Cnr2* cKO mice had reduced tail-flick sensitivity compared to MOR KO, DAT-*Cnr2* cKO, and wild-type controls. These findings suggest that MOR deletion and cell-specific loss of CB2Rs in dopamine neurons or microglia distinctly affect nociceptive functions, alongside immune signaling changes. Targeting components of the eCBome and opioid systems may offer novel therapeutic strategies for inflammation-linked chronic pain.

## Introduction

Recent evidence indicates that opioid and cannabinoid receptors may interact functionally within the central nervous system (CNS). These interactions can occur directly, such as through receptor heteromerization, or indirectly via signaling convergence, in which activation of one system influences endogenous ligand release or downstream signaling in another. Such receptor interplay may underlie various behavioral effects associated with drug use, including acute antinociception [1]. The opioid receptor system includes three main subtypes—mu (MOR), delta (DOR), and kappa (KOR)—which are activated by endogenous peptides such as β-endorphin, enkephalin, and dynorphin [2]. In parallel, the endocannabinoid system (ECS) consists of two classical cannabinoid receptors (CB1Rs and CB2Rs), endogenous cannabinoids (eCBs), and the enzymes that synthesize and degrade them. The discovery of additional lipid mediators, enzymes, and receptors has broadened this system into what is now termed the endocannabinoidome (eCBome) [3].

CB2Rs are predominantly expressed in peripheral tissues, particularly within organs involved in immune function [4, 5]. They are also present in central nervous system cells, including microglia and neurons in the hippocampus, striatum, and brainstem [6]. Although the functional presence of CB2Rs in neurons has been debated, mounting evidence—including our own research—has confirmed their expression and regulatory role in response to drugs of abuse [7–11]. Opioid and cannabinoid receptors share similar signaling characteristics. As G protein-coupled receptors (GPCRs), they inhibit cyclic AMP production via Giα coupling, activate MAP kinase pathways through secondary messengers, and suppress neurotransmitter release by inhibiting calcium influx and promoting potassium efflux [12, 13]. Opioid and cannabinoid receptors share similar Gi-coupled signaling properties, and their effects may converge at the level of intracellular pathways or neuroimmune modulation; however, the present study does not test direct receptor–receptor interactions [14].

There is growing evidence that the opioid and endocannabinoid systems interact across molecular and behavioral domains, influencing processes such as pain perception, reward mechanisms, and addiction susceptibility [15, 16]. Recognizing this interplay may inform the development of novel therapeutics with reduced risk of abuse. Strategically targeting both systems holds promise for advancing treatment approaches for pain and substance use disorders. In behavioral studies involving mice, the endocannabinoid system—particularly CB2Rs—has been implicated in regulating inflammation and motor activity. CB2Rs primarily exert their effects through immune cells and CNS microglia. Their activation has been shown to lower pro-inflammatory cytokine levels and limit immune cell infiltration into affected tissues [3, 17] These receptors also modulate neuroinflammation-related motor function. For instance, in a Parkinson’s disease model, CB2R activation reduced alpha-synuclein aggregation and protected dopaminergic neurons in the substantia nigra, indicating a neuroprotective capacity [17].

Mu-opioid receptors (MORs), the principal targets of endogenous β-endorphin and analgesics such as morphine and fentanyl, play a central role in pain modulation and motor control [18]. MOR activation suppresses neurotransmitter release and induces neuronal hyperpolarization, thereby dampening pain signals. Opioid drugs can also impact locomotor activity in mice—typically enhancing it at low doses via MOR activation. However, higher doses or specific opioid types may instead cause sedation and reduced mobility. These effects can vary based on factors such as drug type, dosage, mouse strain, and co-administered substances [19].

Despite evidence that opioid and cannabinoid systems interact at molecular and behavioral levels, it remains unclear how MOR deletion compares with cell-type-specific CB2 receptor loss in shaping nociceptive behavior and neuroimmune signaling. In particular, the distinct contributions of microglial versus dopaminergic CB2 receptors to pain processing have not been systematically examined. Therefore, the present study aimed to determine how MOR KO, DAT-*Cnr2* cKO, and CX3CR1-*Cnr2* cKO mice differ in nociceptive responses and cytokine expression across the prefrontal cortex and cerebellum. We hypothesized that MOR deletion would produce a pro-inflammatory cytokine profile and altered nociception, whereas microglial and dopaminergic CB2 deletion would yield distinct behavioral and neuroimmune phenotypes reflecting their divergent cellular roles.

## Methods

### Animals

In this study, we utilized MOR KO, DAT-*Cnr2*, and Cx3Cr1-*Cnr2* conditional knockout (cKO) mice, which were generated in our laboratory [20]. These mice were produced through a breeding strategy that involved Cnr2-floxed mice crossed with DAT-*Cre* and Cx3-*Cre* lines. The specific deletion of CB2 receptors (CB2Rs) in dopaminergic neurons and microglia of homozygous cKO mice was confirmed using genotyping and RNAscope *in situ* hybridization. In our previous study, we compared the performances of CB2R-floxed and DAT-*Cre* mice with their C57BL/6J wild-type background controls. Because these groups did not differ significantly in open-field or wheel-running behavior, we used C57BL/6J littermates as the wild-type controls in the present study as well [20]. For behavioral testing, each genotype group consisted of 5 mice and for cytokine analyses, 3 mice per genotype per brain region were included. The number of animals used in our experiments is consistent with prior peer-reviewed studies employing groups of four to five mice and these sample sizes are widely accepted in the field [21–26]. Animals were assigned to experimental order using simple randomization, and all behavioral assessments were performed by experimenters blinded to genotype, as cage labels were coded by an independent laboratory member. Genotypes were not revealed until all behavioral and cytokine measurements were completed. Samples were only omitted if tissue integrity was compromised during dissection or homogenization; no such cases occurred.

All experiments were conducted using adult male mice weighing between 20 and 30 g, bred in the animal facility at William Paterson University of New Jersey. The animals were housed under standardized conditions, including a temperature-controlled environment (25 °C ± 2 °C), a 12:12 h light-dark cycle, and had free access to food and water. All procedures were carried out in accordance with the Guide for the Care and Use of Laboratory Animals and approved by the William Paterson University Institutional Animal Care and Use Committee (IACUC).

### Tail-flick test

The tail-flick test was used to evaluate latency in response to a thermal nociceptive stimulus. Radiant heat was applied approximately 4 cm from the tail tip using a tail-flick apparatus (UGO Basile). Tail-flick latency was recorded as the interval between heat onset and the mouse’s tail withdrawal—an indicator of discomfort triggered by the stimulus. To prevent tissue damage, a cut-off time of 12 s was implemented [20]. Behavioral assessments were conducted by experimenters blinded to genotype. Cage labels were coded by an independent lab member, and genotypes were not revealed until all behavioral scoring was completed.

### Cytokine assay

After the behavioral experiment, the animals were euthanized via decapitation, and their brains were promptly extracted. To facilitate dissection, brains were immediately flash-frozen in liquid nitrogen. Brain regions encompassing the prefrontal cortex (PFC) and cerebellum were isolated and lysed in a cell lysis buffer. Tissue homogenization was carried out using an ultrasonic homogenizer, followed by centrifugation at 10,000 RPM for 5 min to remove cellular debris. The supernatant was collected, protein concentrations were quantified, and the samples were subsequently stored at −80 °C pending cytokine analysis. To evaluate the expression levels of cytokines (IL-1α, IL-1β, IL-6, IL-10, IFN-γ), and chemokines (CXCL10), a Mouse Inflammation ELISA Strip Kit (Signosis, Sunnyvale, CA, USA) was employed. In brief, 100 μL of diluted cell lysate was added to wells pre-coated with primary antibodies specific to each cytokine. After a 1-h incubation at room temperature, the wells were aspirated and washed three times with 200 μL of assay wash buffer. Next, 100 μL of a biotin-conjugated antibody mix was added and incubated for an additional hour under the same conditions, followed by another set of washes. Subsequently, 100 μL of streptavidin-HRP conjugate was added and incubated for 45 min. After a final wash cycle, 100 μL of substrate solution was introduced and incubated for 10 min, after which 50 μL of stop solution was added to terminate the reaction. Absorbance was measured at 450 nm using a microplate reader [3, 27]. All ELISA measurements were performed in duplicate, and mean absorbance values were used for statistical analysis.

### Statistical analysis

Data are presented as mean ± SEM. Sigma Plot 12.0 statistical program was used. Prior to performing the tests, we conducted a normality test (Shapiro-Wilk) to verify the distribution of the data. All datasets met ANOVA assumptions based on Shapiro–Wilk normality testing. The statistical analysis was performed by the one-way and two-way analysis of variance (ANOVA). Post hoc comparisons of means were carried out with Tukey’s test for multiple comparisons when appropriate. Data from the behavioral study were analyzed using one-way ANOVA. We used two-way ANOVA for the analysis of cytokine assay data. One of the factors of the ANOVA was the genotype (MOR KO, DAT-*Cnr2*, Cx3Cr1-*Cnr2* or WT mice) and the other factor was brain region (prefrontal cortex or cerebellum). For all cytokine analyses, Tukey’s *post hoc* test was applied following significant main effects or interactions in the two-way ANOVA. The confidence limit of *p* < 0.05 was considered statistically significant.

## Results

### Tail flick test

**FIGURE 1:**
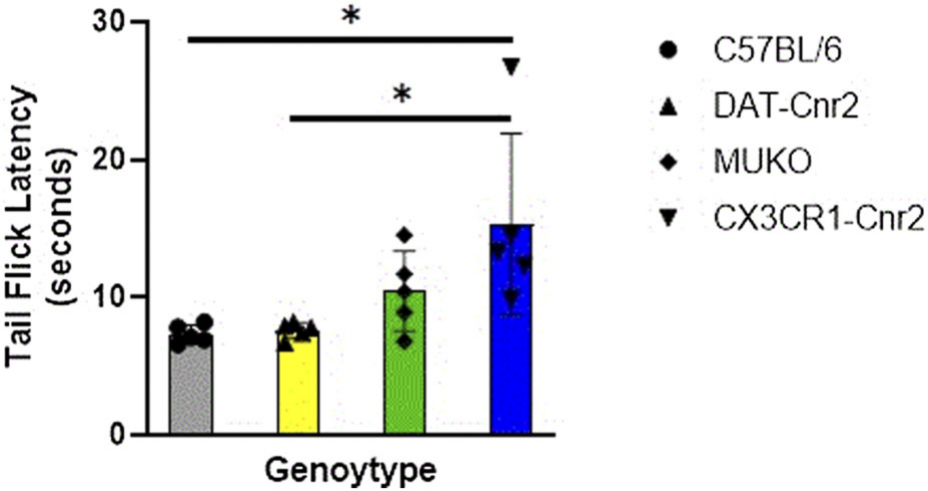
Tail Flick Latency Test in C57BL/6 WT, DAT-*Cnr2*, Cx3Cr1-*Cnr2* and MOR KO Mice. A focused radiant heat source was applied to the tail, and latency to tail withdrawal was recorded. Data represent mean ± SEM for each genotype (n = 5 per group). Statistical significance was determined using one-way ANOVA with *post hoc* Tukey’s multiple comparisons. *p < 0.05, compared to C57BL/6-WT group.

Figure 1 presents a comparative analysis of tail flick latency - an indicator of pain sensitivity - across four different genotypes: C57BL/6, DAT-*Cnr2*, MOR KO, and CX3CR1-*Cnr2*. Tail flick latency measures the time it takes for a mouse to react to a painful stimulus, radiation in this study. The results showed significant main effect for genotype (*F*_(3, 16)_ = 3.611, *p* < 0.05). Post-hoc analysis using Tukey’s test for multiple comparisons revealed that deletion of CB2 receptors from microglia in CX3CR1-*Cnr2* mice significantly (*p* < 0.05) increased tail flick latency compared with the wild type C57BL/6 and DAT-*Cnr2* group. The MOR KO genotype also shows elevated tail flick latency, though not as statistically significant as CX3CR1-Cnr2 group. Altogether, the results underscore the impact of targeted Cnr2 expression in various cell types on altering pain perception. CX3CR1-*Cnr2* mice demonstrate an increased tail-flick latency, indicating reduced nociceptive sensitivity consistent with enhanced antinociception, pointing to potential avenues for exploring cannabinoid receptor pathways in managing pain.

### Cytokine assay

**FIGURE 2:**
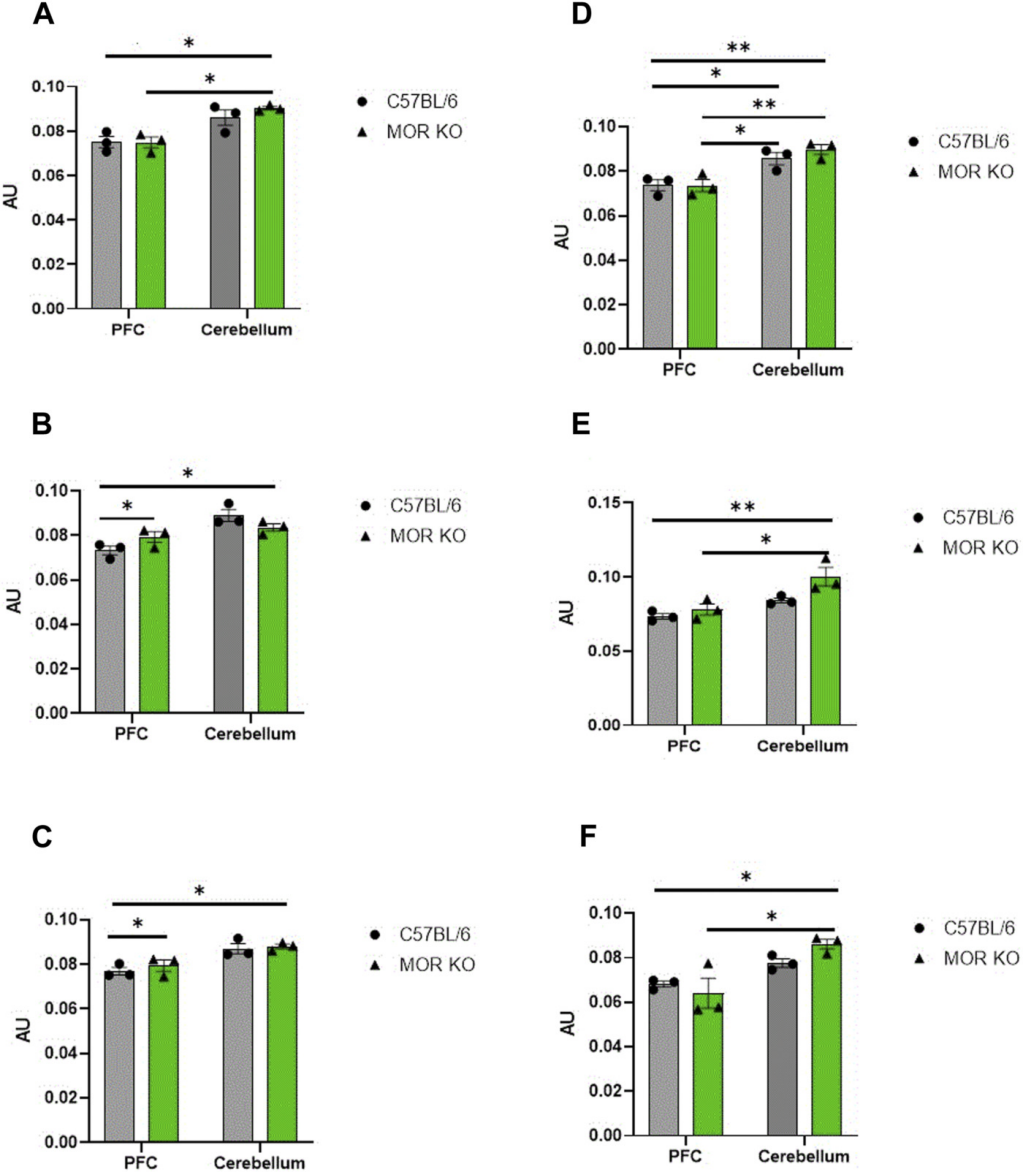
Measures of the levels of proinflammatory cytokines IL-1α **(A)**, IL-1β **(B)**, IL-6 **(C)**, IFN-γ **(D)**, IL-10 **(E)**, and chemokine CXCL-10 **(F)** in the PFC and cerebellum of C57BL/6 WT, DAT-*Cnr2*, Cx3Cr1-*Cnr2* and MOR KO mice. Statistical analysis was done using Two-way ANOVA followed by Tukey’s multiple comparisons test. Values are mean ± SEM (n = 3 in each group). **p < 0.01, *p < 0.05. AU–absorbance unit.

Although only cytokines showing significant genotype or region effects are highlighted in the study, all cytokines in the assay panel were analyzed across all genotypes and brain regions. Cytokines that did not show statistically significant differences are not reported here. The results from the cytokine study showed brain region and genotype significantly affected the expression of IL-1α [brain region effect: *F*_(1, 8)_ = 27.53, *p* < 0.001], IL-1β [brain region effect: *F*_(1, 8)_ = 20.16, *p* < 0.01; brain region X genotype interaction: *F*_(1, 8)_ = 6.628, *p* < 0.05], IL-6 [brain region effect: *F*_(1, 8)_ = 22.69, *p* < 0.01], IFN-γ [brain region effect: *F*_(1, 8)_ = 29.98, *p* < 0.01], IL-10 [brain region effect: *F*_(1, 8)_ = 18.02, *p* < 0.01; genotype effect: *F*_(1, 8)_ = 7.2, *p* < 0.05], and CXCl-10 [brain region effect: *F*_(1, 8)_ = 17.95, *p* < 0.001]. Compared to the C57BL/6, Tukey’s test revealed that there was statistically significant increase (*p* < 0.05) in the levels of IL-β and IL-6 as evidenced by enhanced absorbance values, in MOR KO mice. There is also statistically significant (*p* < 0.05) difference in the expression of IL-1α, IFN-γ, IL-10, and CXCl-10 between cerebellum and PFC of MOR KO mice (Figure 2).

## Discussion

Cannabinoid CB2 and MORs play distinct yet complementary roles in pain modulation through neuroimmune and neuronal mechanisms. CB2 receptors have emerged as promising targets for analgesia without the psychotropic side effects associated with CB1 activation. In chronic pain states, CB2 expression is upregulated in activated microglia, where it modulates inflammatory responses and dampens nociceptive signaling within the spinal cord. Selective CB2 agonists have demonstrated efficacy in reducing pain and inflammation in preclinical models, suggesting their therapeutic potential for neuropathic pain [28, 29]. MORs are widely distributed throughout the central and peripheral nervous systems and are the principal mediators of opioid-induced analgesia. Activation of MORs inhibits nociceptive transmission by suppressing neurotransmitter release and hyperpolarizing neurons via G-protein-coupled mechanisms [30–32]. Interestingly, endogenous opioids and cannabinoids may interact synergistically, with evidence indicating that CB2 activation can modulate opioid receptor signaling and *vice versa*, offering a potential avenue for combination therapies that enhance analgesia while minimizing adverse effects [33]. Together, these receptor systems represent critical nodes in the pain circuitry and are central to the development of next-generation analgesics. Although prior studies have reported synergistic behavioral effects when MOR and CB2 pathways are co-activated, the present study does not investigate molecular interactions between these receptors.

The observed increase in tail flick latency in CX3CR1-*Cnr2* mice underscores the critical role of CB2 receptors in microglia in modulating nociceptive responses. CB2 receptors are known to exert anti-inflammatory and neuroprotective effects, and their deletion has been shown to enhance pain sensitivity by disrupting microglial homeostasis [3, 20, 27, 34]. The significant elevation in latency compared to both wild-type C57BL/6 and DAT-*Cnr2* mice suggests that microglial CB2 signaling plays a more pivotal role in thermal nociception than CB2 expression in dopaminergic neurons [35, 36]. Although CX3CR1-*Cnr2* mice displayed significantly increased tail-flick latency, this finding should not be interpreted exclusively as enhanced antinociception. Tail-flick behavior is a spinally mediated reflex that can be modulated by factors beyond nociceptive threshold, including motor responsiveness, stress reactivity, and microglial influences on sensorimotor integration. Given that CB2 receptors regulate microglial activation and cytokine signaling, the altered latency may reflect broader neuroimmune effects rather than a pure analgesic phenotype.

Interestingly, MOR KO mice also exhibited elevated latency, albeit less pronounced. This aligns with previous studies indicating that MORs are central to opioid-mediated analgesia, and their absence leads to altered pain thresholds [19, 37]. However, the differential magnitude of latency between MOR KO and CX3CR1-*Cnr2* mice may reflect distinct mechanisms - with MOR deletion affecting opioid signaling pathways, and CB2 deletion influencing neuroimmune interactions. These findings support the hypothesis that microglial CB2 receptors and MORs independently contribute to nociceptive regulation, and their disruption leads to enhanced antinociceptive responses, possibly via compensatory mechanisms involving cytokine signaling or altered neurotransmission.

The cytokine profile reveals a complex interplay between genotype and brain region in regulating neuroinflammation. Extensive evidence supports the involvement of both the prefrontal cortex and cerebellum in pain modulation, in addition to their roles in other behavioral processes [38–42]. Meta-analysis of studies employing experimental pain stimuli indicates the following brain areas to be positively associated with pain: primary and secondary somatosensory cortices, insular cortex, ACC, PFC, and thalamus [43]. The cerebellum plays a significant role in pain processing, particularly in inhibiting pain sensation Activation in lobules I–VI, VIII, and the vermis has been linked to nociceptive signalling, fear conditioning, and sensory integration [44]. The significant upregulation of IL-1β and IL-6 in MOR KO mice compared to C57BL/6 suggests a pro-inflammatory shift, consistent with prior reports that MOR deletion leads to enhanced glial activation and cytokine release [45, 46]. IL-1β and IL-6 are key mediators of neuroimmune responses, and their elevation may reflect compensatory immune activation in the absence of MOR-mediated inhibitory signaling.

Moreover, the regional differences in cytokine expression - particularly IL-1α, IFN-γ, IL-10, and CXCL-10 between the cerebellum and PFC - highlight the spatial heterogeneity of neuroimmune regulation. CXCL-10, an IFN-γ-inducible chemokine, is known to recruit T cells and modulate neuroinflammation, and its differential expression may indicate region-specific immune surveillance or vulnerability [47]. The increase in IL-10, an anti-inflammatory cytokine, in specific regions may represent a homeostatic counterbalance to elevated pro-inflammatory signals. This dynamic is supported by literature showing that cytokine expression in the brain is shaped by neural activity and regional microglial phenotypes [48]. Although the current study did not include a formal correlation analysis between cytokine levels and behavioral outcomes, the cytokine profile of MOR KO mice—characterized by elevated IL-1β and IL-6—suggests a pro-inflammatory shift that may contribute to altered nociceptive processing. In contrast, CX3CR1-Cnr2 mice exhibited increased tail-flick latency without a parallel increase in these cytokines, indicating that microglial CB2 deletion may influence nociception through mechanisms other than classical pro-inflammatory cytokine upregulation. Because the Brief Communication format restricts the amount of data that can be presented, only cytokines with significant effects are discussed here; however, all measured cytokines were analyzed across genotypes and brain regions.

This study has several strengths, including the use of multiple genetically modified mouse lines that allow for cell-type-specific dissection of CB2 receptor function, as well as direct comparison with MOR deletion. By integrating behavioral measures with cytokine profiling across two brain regions, we provide a multidimensional view of how MOR and CB2 signaling influence nociceptive and neuroimmune processes. However, nociception was assessed using only the tail-flick test, which primarily measures spinal reflexive withdrawal; additional assays such as hot-plate, von Frey, or Hargreaves would help determine whether the observed effects generalize to supraspinal or mechanical modalities. The cytokine analyses were conducted using small group sizes, which increases the risk of both Type I and Type II errors. Although these sample sizes are consistent with prior neuroimmune studies using mouse brain tissue, the findings should be interpreted cautiously and considered preliminary given the small sample size and limitations of normality testing. Larger, adequately powered studies will be necessary to confirm the genotype-specific immune patterns observed here.

## Conclusion

In conclusion, the current findings provide compelling evidence that both cannabinoid CB2 and mu opioid receptors contribute to distinct behavioral and neuroimmune phenotypes. The significant increase in tail flick latency observed in CX3CR1-*Cnr2* and MOR KO mice underscores their critical role in antinociceptive regulation, with CB2 deletion from microglia exerting a more pronounced effect. Concurrently, the cytokine data highlight genotype- and region-specific immune dynamics, with MOR KO mice showing elevated pro-inflammatory cytokine levels and the cerebellum and PFC exhibiting differential immune profiles. Together, the finding deepens our understanding of how neuroimmune interactions contribute to pain physiology and support the therapeutic potential of targeting these receptors to develop more effective and selective analgesics.

## Data Availability

The original contributions presented in the study are included in the article/supplementary material, further inquiries can be directed to the corresponding authors.
